# Supplementary material for: Cell type-dependent differential activation of ERK by oncogenic KRAS in colon cancer and intestinal epithelium
Source: Nat Commun. 2019 Jul 2;10:2919. doi: 10.1038/s41467-019-10954-y (PMC6606648; doi:10.1038/s41467-019-10954-y)
Supplement: Supplementary file 1 — Supplementary Information [file 41467_2019_10954_MOESM1_ESM.pdf]

Cell type-dependent activation of ERK by oncogenic KRAS in colon cancer and intestinal epithelium

Brandt and Sell *et al.*

Supplementary Figures 1-9

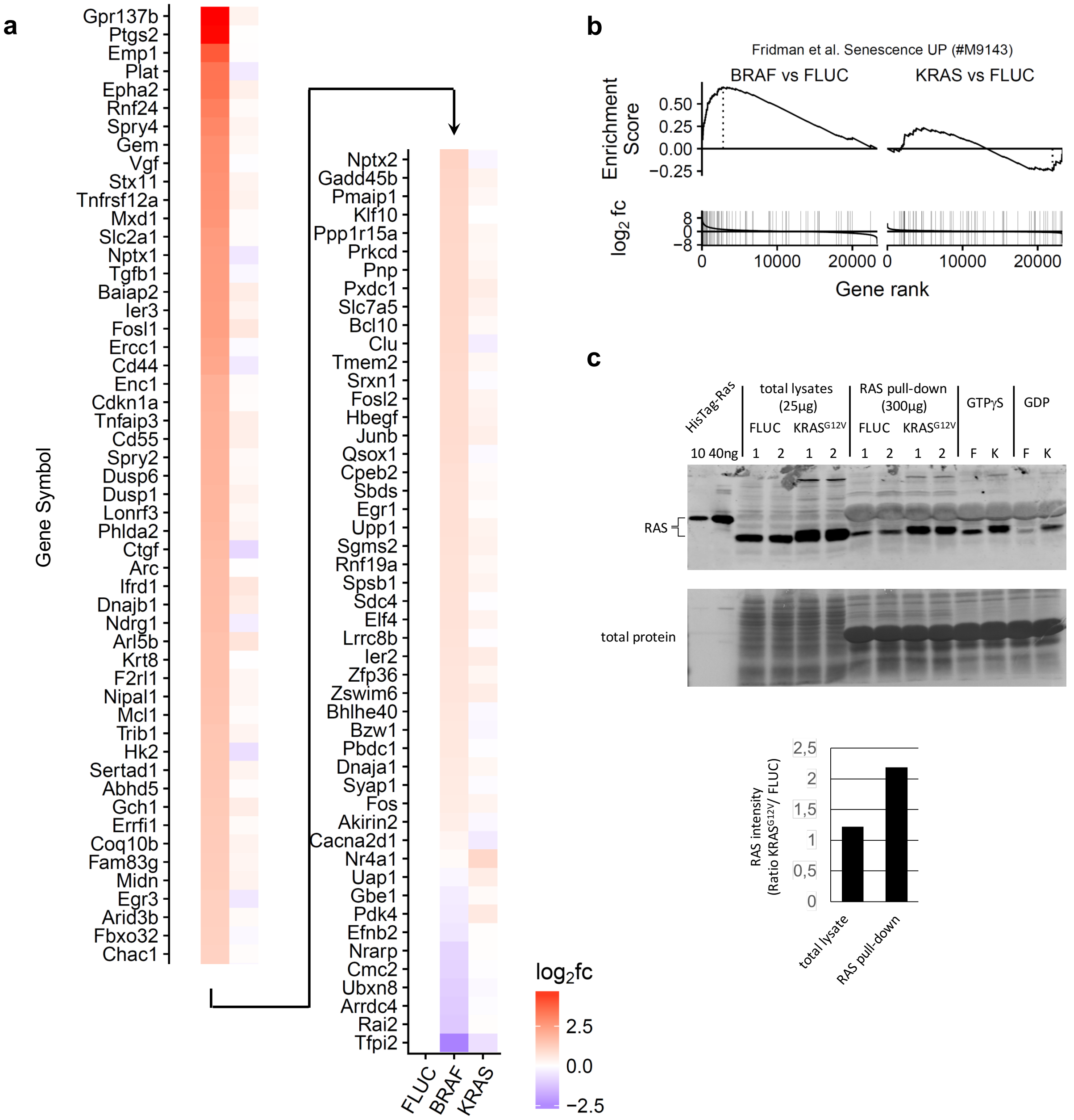

Supplementary Figure 1: Bulk tissue transcriptome analyses of KRAS^{G12V} and BRAF^{V600E}-induced organoids and KRAS activity assays. a) MAP kinase target genes, as defined in ref. 1 were assessed in transcriptomes 24 h after transgene induction. Near universal upregulation was found after BRAF^{V600E}, but not after KRAS^{G12V}, induction. b) BRAF^{V600E}, but not KRAS^{G12V} induces a senescence-associated gene signature. Replicate transcriptomes were generated from transgene-positive cells of induced organoids, as described before [2]. RNA-seq data are available in under accession number GSE93947. c) Transgenic KRAS supplements total RAS activity. Panels show RAS western blot (top) and total protein (bottom). Quantification of RAS is shown below. Experiments were done in two biological replicates, indicated by numbers 1 and 2.

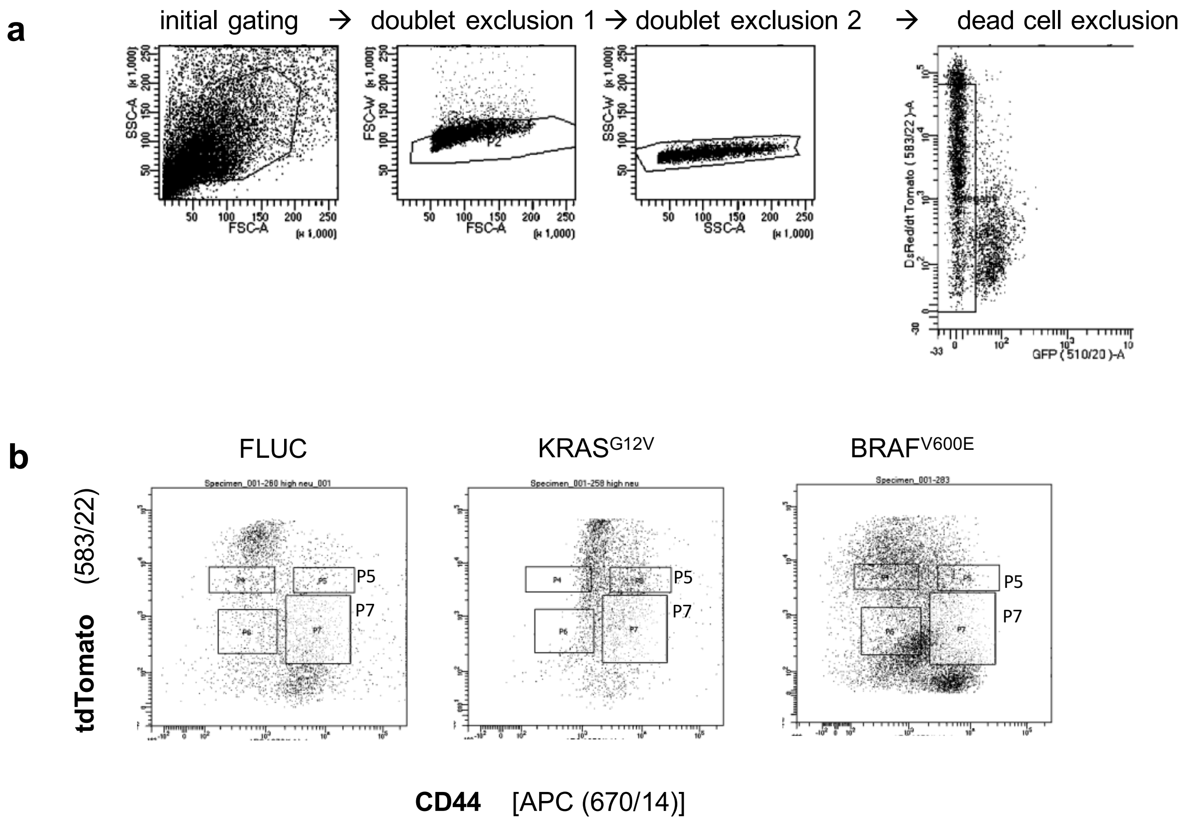

Supplementary Figure 2: FACS gating strategy for FLUC-, KRAS^{G12V}-, and BRAF^{V600E} cells for single cell sequencing. A) Gating strategy to obtain alive single cells. B) Representative example of tdTomato (transgene; 583/22 bandpass (BP) filter) and CD44 (670/14 BP filter) fluorescence in transgene-induced organoid cell suspensions, as indicated. Note that induction of KRAS^{G12V}, but not of BRAF^{V600E}, increases levels of crypt cell marker CD44. This effect is likewise seen in the CyTOF data sets (compare Fig. 6E). Gate P5 was used to sort KRAS^{G12V}-induced cells. Gate P7 was used to sort for BRAF^{V600E}-induced cells. The alternative gate (as compared to KRAS^{G12V} cells) was used, as high BRAF^{V600E} expression results in intestinal cell death (see Fig. 2 and ref. 3). Both gates, P5 and P7, were employed for FLUC-induced cells.

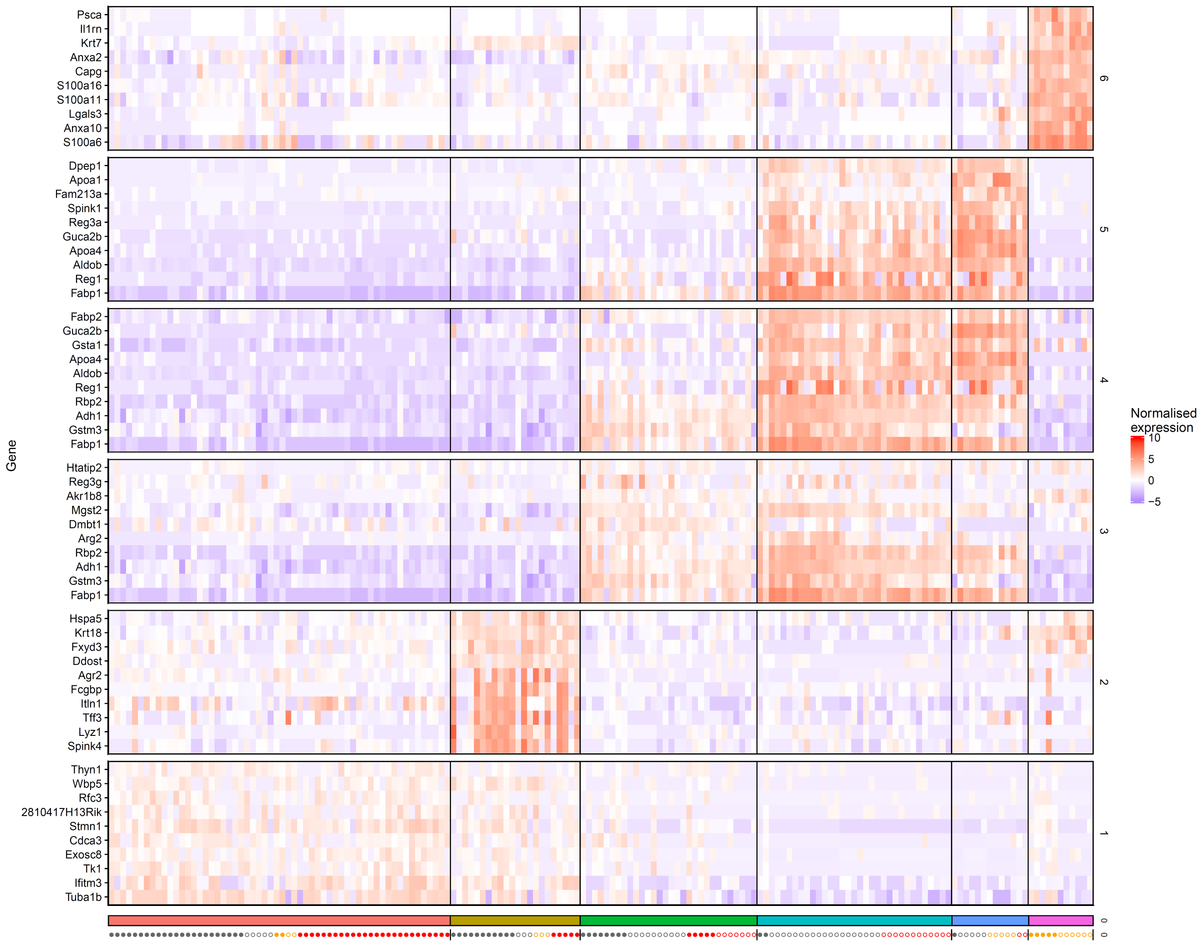

Supplementary Figure 3: Signature genes for six transcriptome clusters derived from FLUC, KRAS^{G12V} and BRAF^{V600E}-induced organoid cells. Top 10 overrepresented genes per cluster are given (bottom to top). Colour code for clusters and single cells (on x-axis) as in Fig. 3 (cells marked in grey: control FLUC transgene induced; red: KRAS^{G12V}-induced; yellow: BRAF^{V600E}-induced; full circle: sorted as CD44-high; empty circle: sorted as CD44-low).

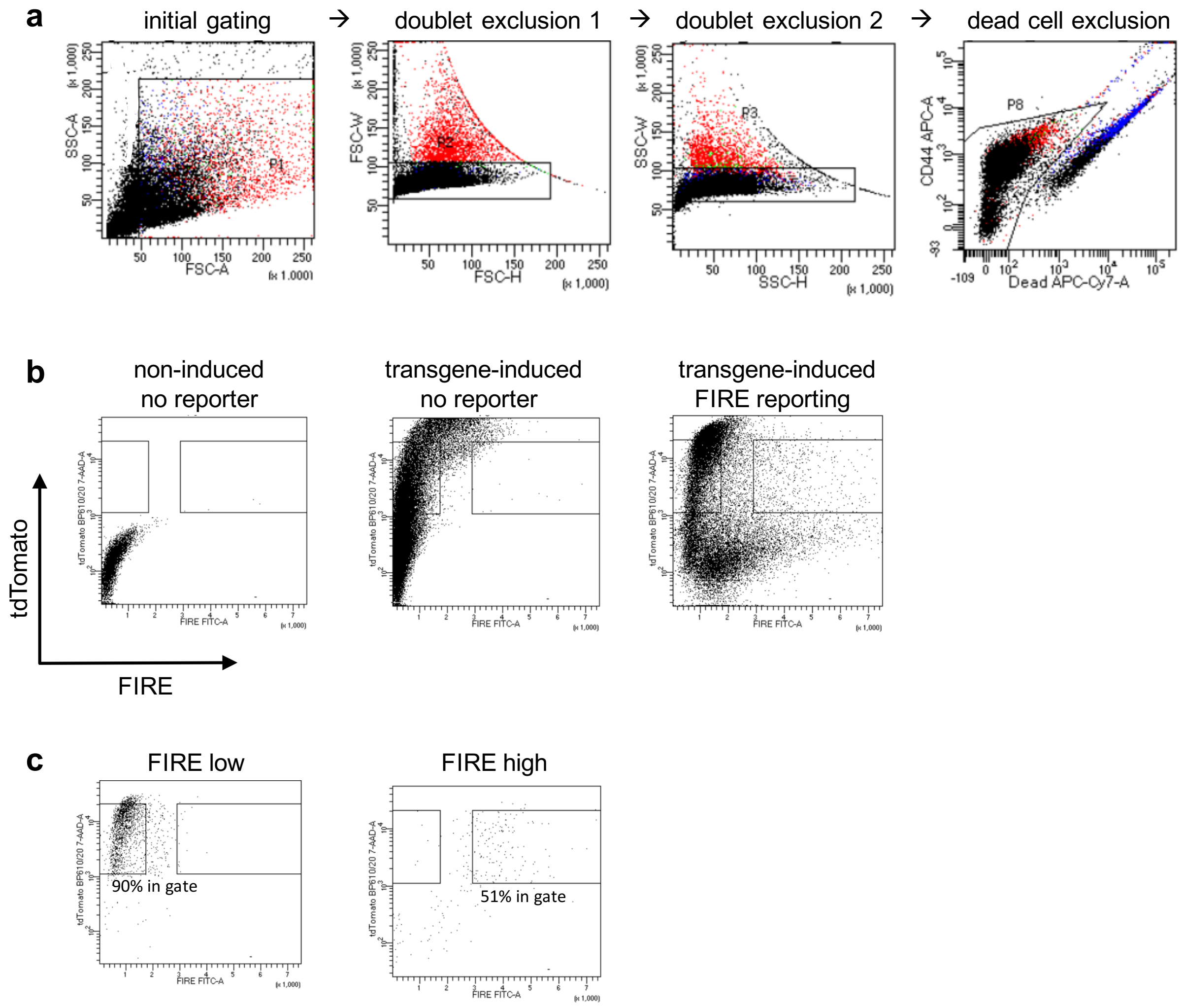

Supplementary Figure 4: FACS gating strategy for transgene induced FIRE-positive and -negative cells for single cell sequencing. a) Gating strategy to obtain alive single cells b) Representative example of tdTomato (transgene; 610/20 BP) and FIRE (ERK reporter; 530/30 BP) fluorescence in control and transgenic organoid cell suspensions, as indicated. FIRE fluorescence is low and detected in linear detector mode c) Post sort analysis for FIRE-positive and negative cells. In short, induced organoid cells were sorted into BSA-coated tubes, using FIRE-negative and FIRE-positive gates, as indicated. Numbers indicate percent of target population from all events.

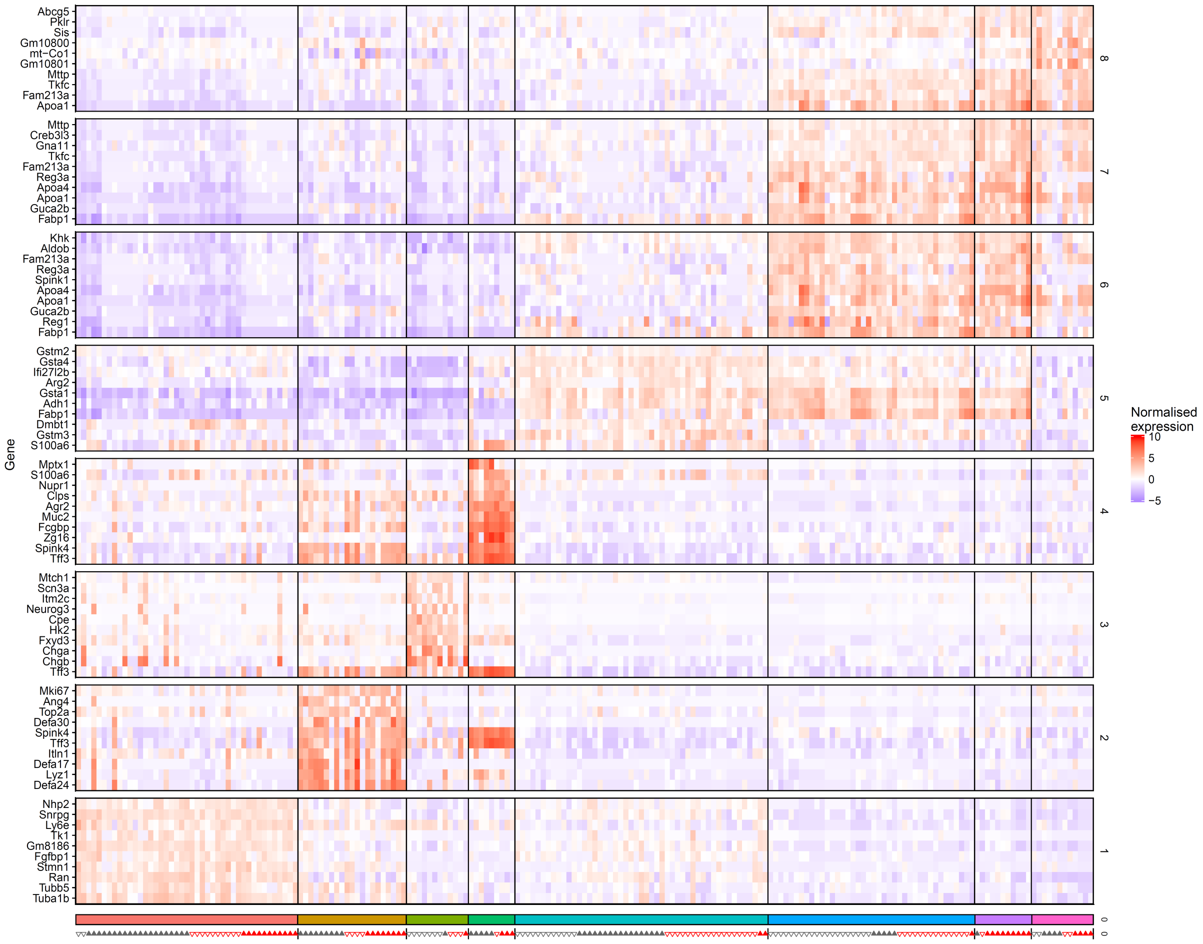

Supplementary Figure 5: Signature genes for eight transcriptome clusters derived from organoid cells sorted by FIRE activity. Top 10 overrepresented genes per cluster are given (bottom to top). Colour code for clusters and single cells (on x-axis) as in Fig. 5 (cells marked in grey: control FLUC transgene-induced; red: KRAS^{G12V}-induced; full arrowhead up: sorted as FIRE positive; empty arrowhead down: sorted as FIRE negative).

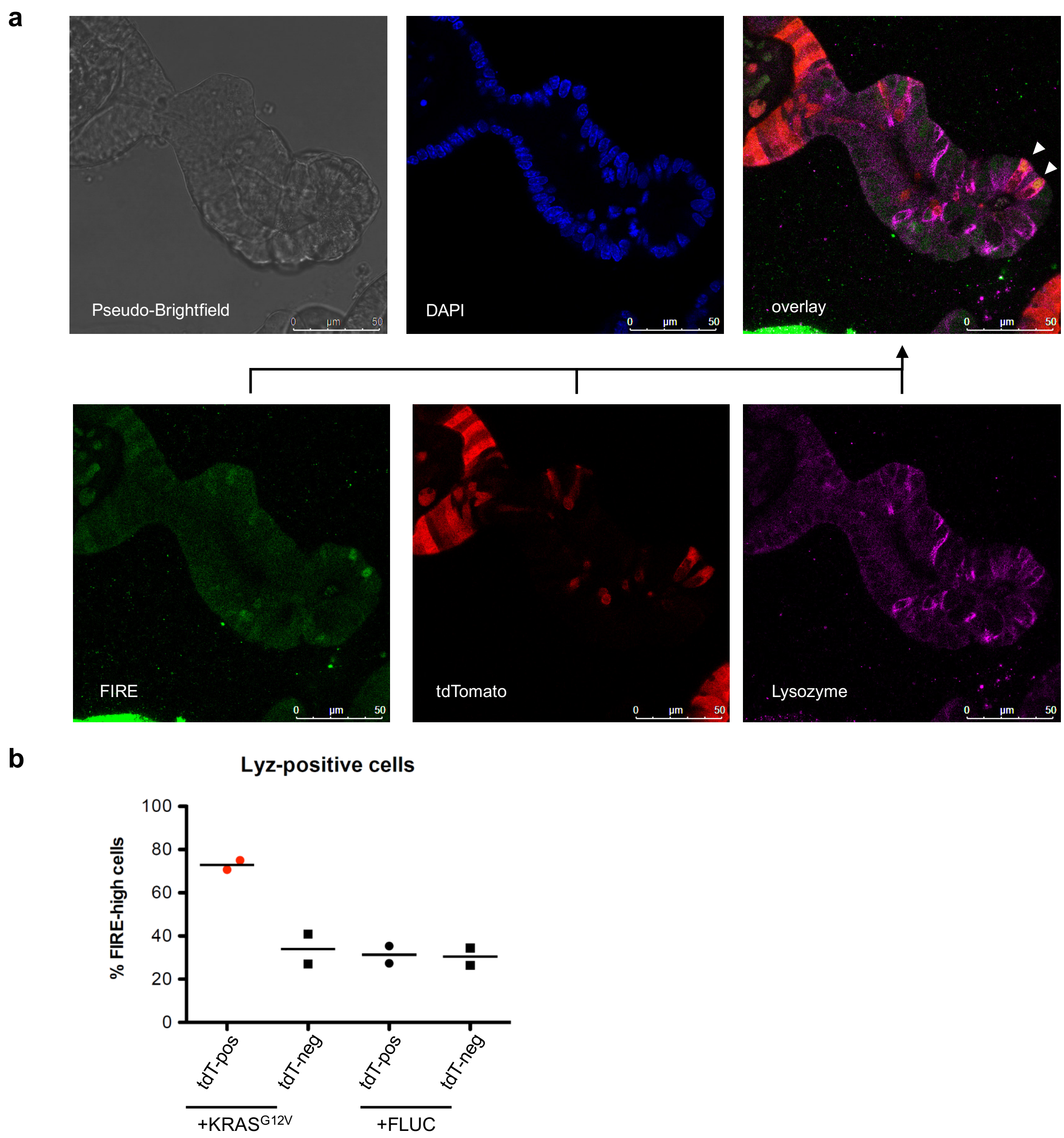

Supplementary Figure 6: Immunofluorescence confirms Paneth cells as a main FIRE-positive cell type in the crypt. a) Pseudo bright field image along with fluorescent channels representing nuclear stain (DAPI; blue), FIRE fluorescence (Venus; green), transgene fluorescence (tdTomato; red), and Paneth cell immunostaining (Lysozyme; purple) are given. Overlay combines the green, red and purple channels. b) Quantification of FIRE-high Lysozyme (Lyz)-positive Paneth cells after induction of $KRAS^{G12V}$- or FLUC-encoding transgenes. Data represents 2 independent experiments with >100 cells Lyz(+) cells counted per condition.

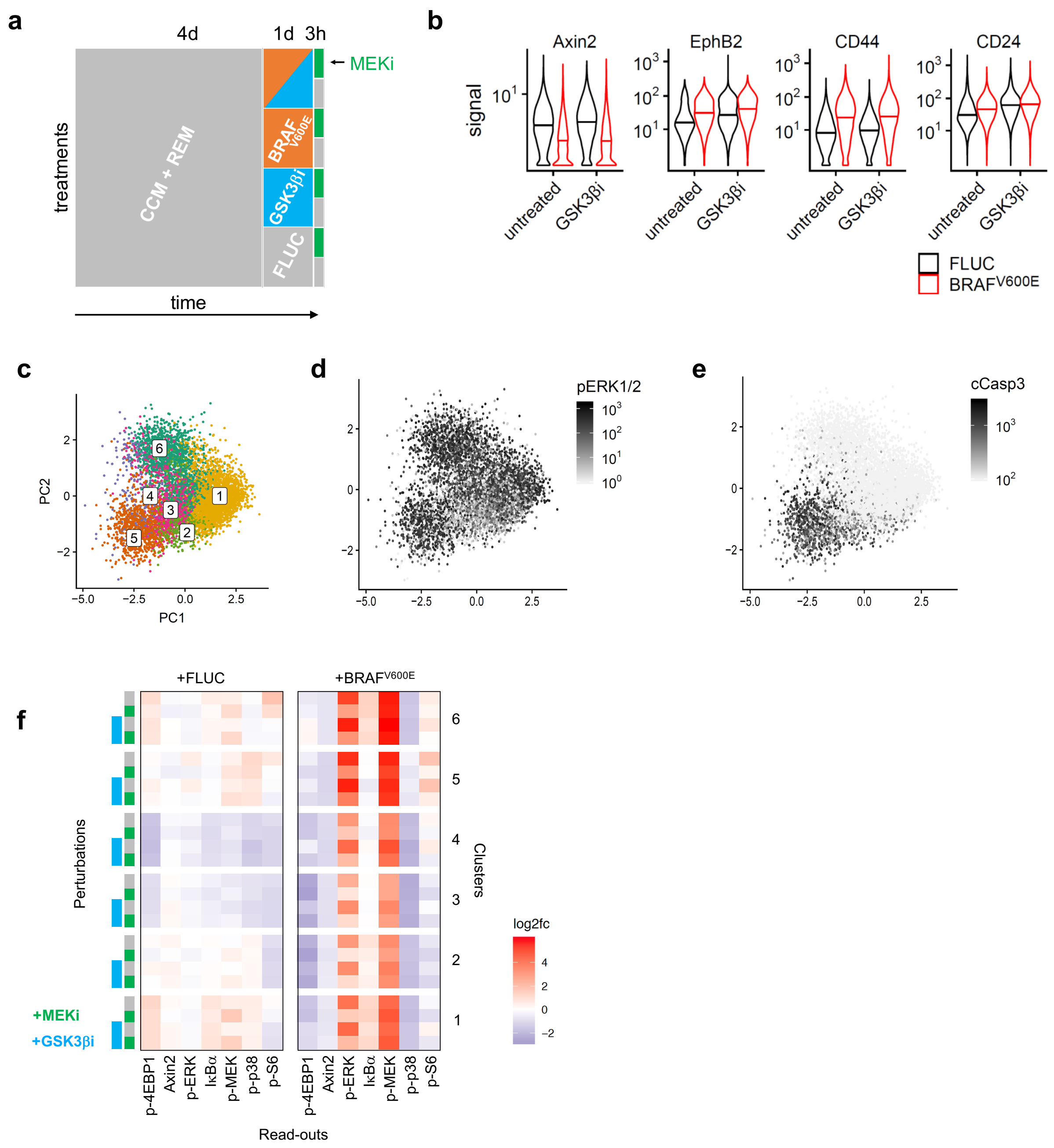

Supplementary Figure 7: CyTOF analysis reveals BRAF^{V600E}-induced changes in signalling networks. a) Schematics for generation of network perturbation data by CyTOF. For details, see legend to Figure 6. b) Distributions of cell type markers in organoid cells induced for FLUC or BRAF^{V600E} transgenes plus/minus GSK3β inhibitor treatment. Central lines of violin plots denote median values. c) PCA showing colour code of k-means clustering in BRAF^{V600E}-induced cells by EphB2, CD44, CD24, Krt20, cleaved Caspase 3 signal strength. Clustering was initially done in FLUC control cells to ensure compatibility with the KRAS^{G12V} experiment in Figure 6. d-e) Mapping of signal strength for p-ERK and cleaved Caspase3 on PCA, as in c). f) Protein phosphorylation or abundance CyTOF data, demultiplexed by treatment and cell clusters, as defined in panel c). CyTOF data given as log2 fold changes to average untreated FLUC-induced control line.

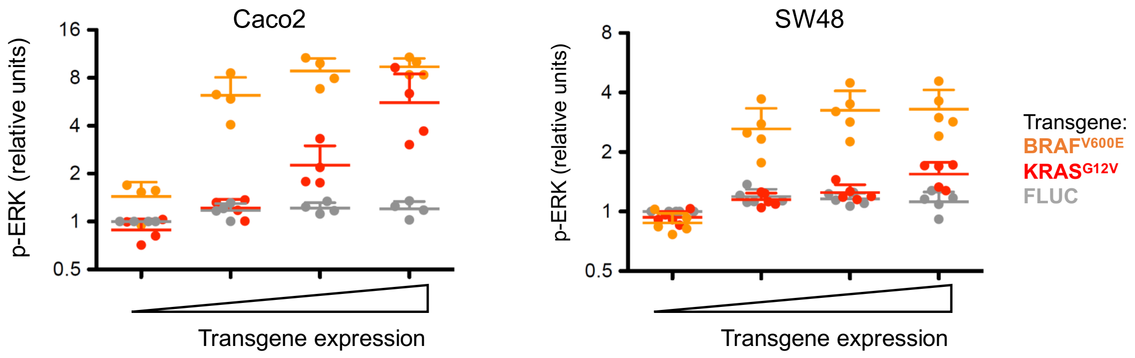

Supplementary Figure 8: Signal transduction from KRAS^{G12V} to ERK differs between CRC cell lines. Quantification of phospho-ERK in KRAS^{G12V}-, BRAF^{V600E}- or FLUC-transfected Caco2 and SW48 CRC cells, by flow cytometry. Mean and standard deviation of 4 independent replicate experiments is given. Grey: FLUC-; orange: BRAF^{V600E}; red: KRAS^{G12V}-transfected cells. SW48 and Caco2 CRC cell lines have no mutations in KRAS, NRAS or BRAF. For the experiments, cells were transfected with inducible expression vectors for tdTomato-KRAS^{G12V}, -BRAF^{V600E}, or -FLUC control, as previously used in organoid cultures. 24 h after induction, cells were fixed and oncogene levels, as extrapolated from tdTomato, and phosphorylation of ERK were measured via flow cytometry. We found that even low levels of BRAF^{V600E} (orange dots) strongly increased ERK phosphorylation in both cell lines. In contrast, the ability of KRAS^{G12V} (red dots) to induce ERK phosphorylation was markedly different. In Caco2 cells, ERK phosphorylation increased stepwisely with KRAS^{G12V}-associated fluorescence, whereas in SW48 even high levels of transgenic tdTomato-KRAS^{G12V} were unable to increase ERK phosphorylation.

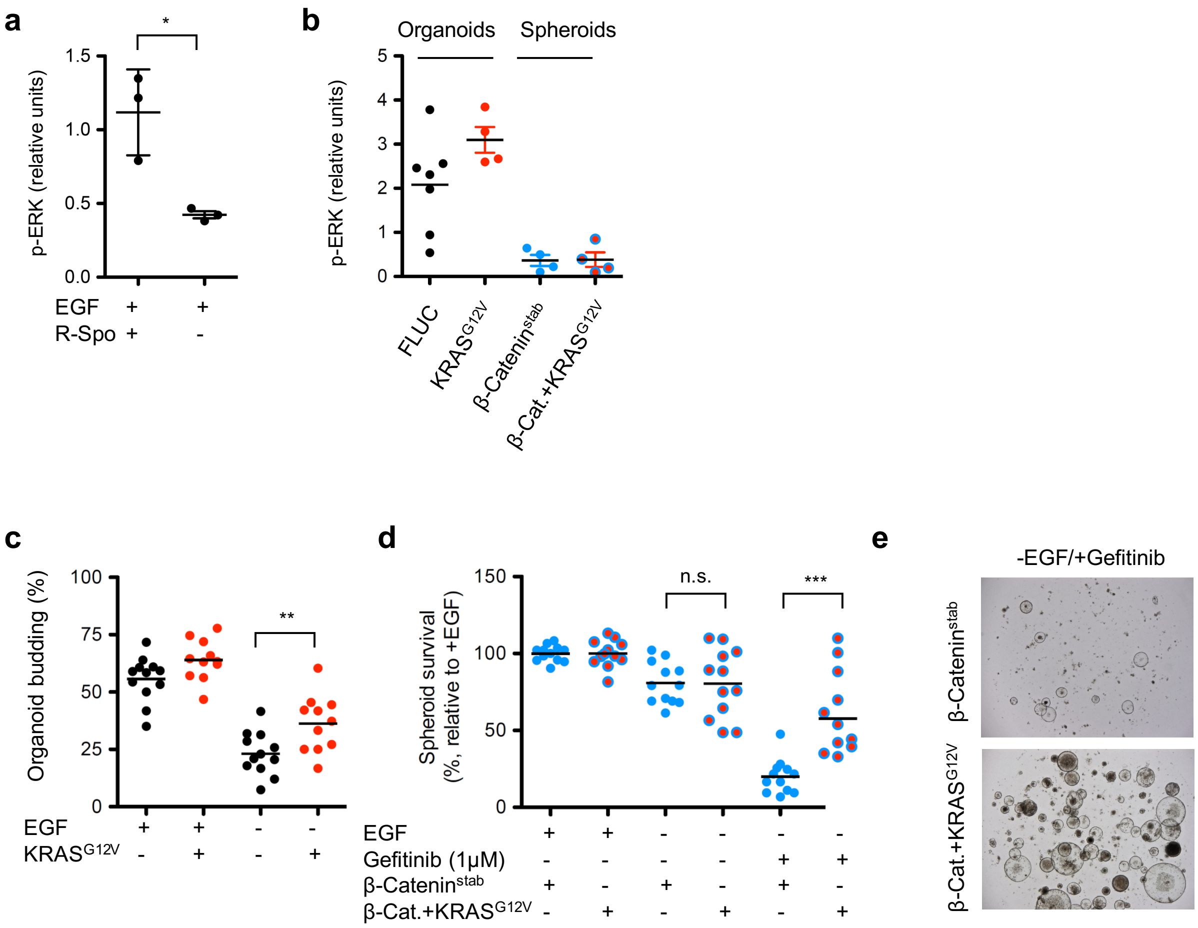

Supplementary Figure 9: Interaction between β-Catenin and RAS-ERK signalling in intestinal organoids. a) p-ERK quantification by capillary protein analysis, 24 h after R-Spondin removal. Results indicate decrease of ERK phosphorylation after abrogation of Wnt/β-Catenin activity in organoids. b) phospho-ERK quantification by capillary protein analysis in spheroid cells that have undergone a switch to an adenoma-like phenotype after long-term activation of stabilized β-Catenin and removal of the Wnt co-ligand R-Spondin. The results indicate decreasing ERK phosphorylation in cultures with constitutively high β-Catenin activity. We propose that the changes of p-ERK levels observed in a) and b) are due to shifts in cell type. c)-e) Functional interaction between $KRAS^{G12V}$ and β-Catenin. c) Quantification of organoid budding 4 days after induction of oncogenic $KRAS^{G12V}$ in the presence or absence of EGF in normal tissue organoids. Cysts forming after organoid disaggregation were counted immediately after passaging. Fractions of budding organoids were calculated at day 4. It is of note that although a significant increase in budding cysts was observed, organoids could never be propagated after passaging in the absence of EGF, not even after induction of $KRAS^{G12V}$. d) Quantification of spheroid survival after EGF depletion, EGFR inhibition by Gefitinib and/or $KRAS^{G12V}$ induction, 6 days after passaging. Quantification from multiple wells of 3 independent experiments e) Representative images of β-$Catenin^{stab}$- or β-$Catenin^{stab}$/$KRAS^{G12V}$ double transgenic spheroid cultures, 6 days after passaging and in EGF-free medium in the presence of 1 µM Gefitinib. Error bars in figures denote standard deviations. p-values are calculated form two-tailed unpaired t-tests in GraphPad prism. *, ** and *** denote p-values <0.05, <0.01 and <0.001, respectively.

Supplementary References

1. Uhlitz, F. *et al.* An immediate–late gene expression module decodes ERK signal duration. *Mol. Syst. Biol.* **13**, 928 (2017).
2. Riemer, P. *et al.* Oncogenic β-catenin and PIK3CA instruct network states and cancer phenotypes in intestinal organoids. *J. Cell Biol.* **216**, 1567–1577 (2017).
3. Riemer, P. *et al.* Transgenic expression of oncogenic BRAF induces loss of stem cells in the mouse intestine, which is antagonized by β-catenin activity. *Oncogene* **34**, 3164–3175 (2015).
