# Supplementary material for: Cell type-dependent differential activation of ERK by oncogenic KRAS in colon cancer and intestinal epithelium
Source: Nat Commun. 2019 Jul 2;10:2919. doi: 10.1038/s41467-019-10954-y (PMC6606648; doi:10.1038/s41467-019-10954-y)
Supplement: Supplementary file 3 — Reporting Summary [file 41467_2019_10954_MOESM3_ESM.pdf]

nature research

Corresponding author(s): Markus Morkel
Nils Blüthgen

Last updated by author(s): 23/5/2019

Reporting Summary

Nature Research wishes to improve the reproducibility of the work that we publish. This form provides structure for consistency and transparency in reporting. For further information on Nature Research policies, see Authors & Referees and the Editorial Policy Checklist.

Statistics

For all statistical analyses, confirm that the following items are present in the figure legend, table legend, main text, or Methods section.

n/a	Confirmed	
☐	☒	The exact sample size (*n*) for each experimental group/condition, given as a discrete number and unit of measurement
☐	☒	A statement on whether measurements were taken from distinct samples or whether the same sample was measured repeatedly
☐	☒	The statistical test(s) used AND whether they are one- or two-sided<br>*Only common tests should be described solely by name; describe more complex techniques in the Methods section.*
☒	☐	A description of all covariates tested
☐	☒	A description of any assumptions or corrections, such as tests of normality and adjustment for multiple comparisons
☐	☒	A full description of the statistical parameters including central tendency (e.g. means) or other basic estimates (e.g. regression coefficient) AND variation (e.g. standard deviation) or associated estimates of uncertainty (e.g. confidence intervals)
☐	☒	For null hypothesis testing, the test statistic (e.g. *F*, *t*, *r*) with confidence intervals, effect sizes, degrees of freedom and *P* value noted<br>*Give P values as exact values whenever suitable.*
☒	☐	For Bayesian analysis, information on the choice of priors and Markov chain Monte Carlo settings
☐	☒	For hierarchical and complex designs, identification of the appropriate level for tests and full reporting of outcomes
☒	☐	Estimates of effect sizes (e.g. Cohen's *d*, Pearson's *r*), indicating how they were calculated

Our web collection on statistics for biologists contains articles on many of the points above.

Software and code

Policy information about availability of computer code

Data collection	not applicable
Data analysis	Software StasNET 1.0.1 is freely available, see ref. 72. Full code is provided as Supplementary HTML file.

For manuscripts utilizing custom algorithms or software that are central to the research but not yet described in published literature, software must be made available to editors/reviewers. We strongly encourage code deposition in a community repository (e.g. GitHub). See the Nature Research guidelines for submitting code & software for further information.

Data

Policy information about availability of data

All manuscripts must include a data availability statement. This statement should provide the following information, where applicable:

- Accession codes, unique identifiers, or web links for publicly available datasets
- A list of figures that have associated raw data
- A description of any restrictions on data availability

scRNA-seq and bulk RNA-seq data are available in the GEO repository under accession numbers GSE115242 and GSE115234, respectively.

Field-specific reporting

Please select the one below that is the best fit for your research. If you are not sure, read the appropriate sections before making your selection.

☒ Life sciences ☐ Behavioural & social sciences ☐ Ecological, evolutionary & environmental sciences

For a reference copy of the document with all sections, see nature.com/documents/nr-reporting-summary-flat.pdf

Life sciences study design

All studies must disclose on these points even when the disclosure is negative.

Sample size	No statistical methodes were used to determine sample sizes
Data exclusions	No data were excluded from any analyses
Replication	CyTOF data in Fig 1 was replicated in two experiments with similar results, data from one replicate is shown.<br>Data points in Fig. 2 are mean plus/minus SD from at two to four replicate cultures<br>Single cell sequencing data in Figures 3 and 5 were not replicated, but main findings were confirmed via follow-up experiments, as detailled in the manuscript.<br>Fluorescent microscopy and immunohistochemistry images from Fig 4 were obtained from at least three cultures each, with the exception of BRAF(V600E)/FIRE-transgenic organoids were data was only obtained in two experiments. Representative images were selected for each panel.<br>CyTOF perturbation study in Figures 6 was performed twice, with similar results. One replicate experiment of approx. 160 000 individual RFP-positive cells was employed for modelling in Figure 7, and the other experiment served to verify the main findings.<br>RAS pulldown assays in Figure S1c were done once with biological duplicates for each condition.<br>Quantification of Paneth Cell FIRE positivity in Figure S6 was done in two replicate experiments, with similar results.<br>CyTOF perturbation study in Figure S7 was performed twice, with similar results. Figures were made from first replicate, and second replicate served to verify the findings.<br>Assessment of KRAS to ERK signal transduction in Caco2 and SW48 cells in Figure S8 was done in at least three replicate expriments per cell line, as indicated.<br>Experiments with KRAS(G12V)-, Beta-Catenin- and double transgenic organoid cultures in Figure S9 were done at least in three replicate experiments, as indicated.
Randomization	Not applicable
Blinding	Investigators were not blinded, with the exception of counting organoid survival in Fig. 1b and e, where organoids were counted after blinding.

Reporting for specific materials, systems and methods

We require information from authors about some types of materials, experimental systems and methods used in many studies. Here, indicate whether each material, system or method listed is relevant to your study. If you are not sure if a list item applies to your research, read the appropriate section before selecting a response.

Materials & experimental systems

n/a	Involved in the study	
☐	☒	Antibodies
☐	☒	Eukaryotic cell lines
☒	☐	Palaeontology
☐	☒	Animals and other organisms
☒	☐	Human research participants
☒	☐	Clinical data

Methods

n/a	Involved in the study	
☒	☐	ChIP-seq
☐	☒	Flow cytometry
☒	☐	MRI-based neuroimaging

Antibodies

Antibodies used	For CyTOF analysis, we used the following pre-conjugated antibodies from Fluidigm:<br>CD24 (for mouse: 150-Nd, 3150009B, for human: 169-Tm, 3169004B),<br>CD44 (for mouse: 162-Dy, 3162030B, for human: 166-Er, 3166001B)<br>cleaved Casp3 (142-Nd, 3142004A),<br>p-H2AX [S139] (147-Sm, 3147016A)<br>p-Akt [S473] (152-Sm, 3152005A)<br>p-p38 [T180/Y182] (156-Gd, 3156002A)<br>Ki67 (162-Dy, 3168007B)<br>IκBα (164-Dy, 3164004A)<br>p-ERK1/2 [T202/Y204] (171-Yb 3171010A)<br>p-S6 [S235/236] (175-Lu, 3175009A).<br>We labelled the following antidodies with the respective metal tags:<br>Axin2 (145-Nd,Abcam, ab32197, 2 µg/ml)<br>p-MEK1/2 [S217/221] (151-Eu, CST, 41G9, 2 µg/ml)<br>EphB2 (158-Gd, BD, 2H9, 2 µg/ml)<br>p-4e-BP1 [T37/46] (170-Er, CST, 236B4, 2 µg/ml)<br>Krt20 (176-Yb, CST, D9Z1Z, 2 µg/ml).

For capillary protein quantification, we used
p-ERK/2(T202/Y204) (1:50; #9101, Cell Signal)
vinculin (1:30; #4650; Cell Signal).

For IHC and IF analysis, we used:
P-ERK (T202/Y204; #4370 CellSignal)
P-MEK (S217/221; #9121 CellSignal),
RFP (1:200; #600-401-379 Rockland)
Lysozyme (1:250; ab108508, Abcam)

Validation

All prelabelled CyTOF antibodies from Fluidigm were previously tested by the manufacturer. In addition, we tested all CyTOF antibodies against surface proteins individually using cell lines reported to be positive, negative or heterogeneous for the respective proteins to confirm the expected patterns. We tested all CyTOF antibodies against signal transducers or pathway targets using cell line-based assays where we activated/inactivated the respective signaling node by serum withdrawal, kinase inhibitors etc.. The CyTOF panel will be described in detail in another manuscript.

Antibodies used for capillary western were tested for clear bands of the expected sizes in different cell lines in our lab. Antibodies for IF and IHC analysis were tested by the manufacturer and in different cell lines with known signal patterns and strengths in our lab.

Eukaryotic cell lines

Policy information about cell lines

Cell line source(s)

SW48, Caco2 were both acquired from ATCC

Authentication

Cell lines are regularly panel sequenced for mutational patterns in 100 cancer driver genes in our lab to confirm identity

Mycoplasma contamination

Cell lines are regularly checked for mycoplasma contamination in our lab, and contaminated cell lines are discarded.

Commonly misidentified lines
(See ICLAC register)

none used

Animals and other organisms

Policy information about studies involving animals; ARRIVE guidelines recommended for reporting animal research

Laboratory animals

Transgenic organoids were derived from transgenic mice originally derived from F1 hybrid Bl6/129S6 embryonic stem cells. For strain maintenance, mice were backcrossed to C57Bl/6J.

Wild animals

not applicable

Field-collected samples

not applicable

Ethics oversight

Transgenic experimentation to generate mouse lines were approved by Berlin authorities LAGeSo (G0185, G0143/14). Experiments described in the present manuscript involve solely killing of mice for organ removal, and no live animal experimentation.

Note that full information on the approval of the study protocol must also be provided in the manuscript.

Flow Cytometry

Plots

Confirm that:

- [x] The axis labels state the marker and fluorochrome used (e.g. CD4-FITC).
- [x] The axis scales are clearly visible. Include numbers along axes only for bottom left plot of group (a 'group' is an analysis of identical markers).
- [ ] All plots are contour plots with outliers or pseudocolor plots.
- [ ] A numerical value for number of cells or percentage (with statistics) is provided.

Methodology

Sample preparation

For FACS sorting, FIRE- and Transgene-positive and/or -negative organoids cells were disaggregated by TrypLE in presence of DNAse I, as detailled in Methods.

Instrument

BD Aria

Software	BD FACS Diva 8.0.1
Cell population abundance	Cell population abundance in FACS is not dicussed in the manuscript, as FACS is solely used for selection of cells for single cell sequencing
Gating strategy	FIRE- and Transgene- positive gates were determined using cells without FIRE reporter and/or without transgene. Gates for CD44-positivity were determined using transgene-negative organoid cells with or without CD44 antibody. See Figures S2 and S4 for gating strategies.

☒ Tick this box to confirm that a figure exemplifying the gating strategy is provided in the Supplementary Information.
