# Supplementary figures and images for: Cell type-dependent differential activation of ERK by oncogenic KRAS in colon cancer and intestinal epithelium
Source: Nat Commun. 2019 Jul 2;10:2919. doi: 10.1038/s41467-019-10954-y (PMC6606648; doi:10.1038/s41467-019-10954-y)

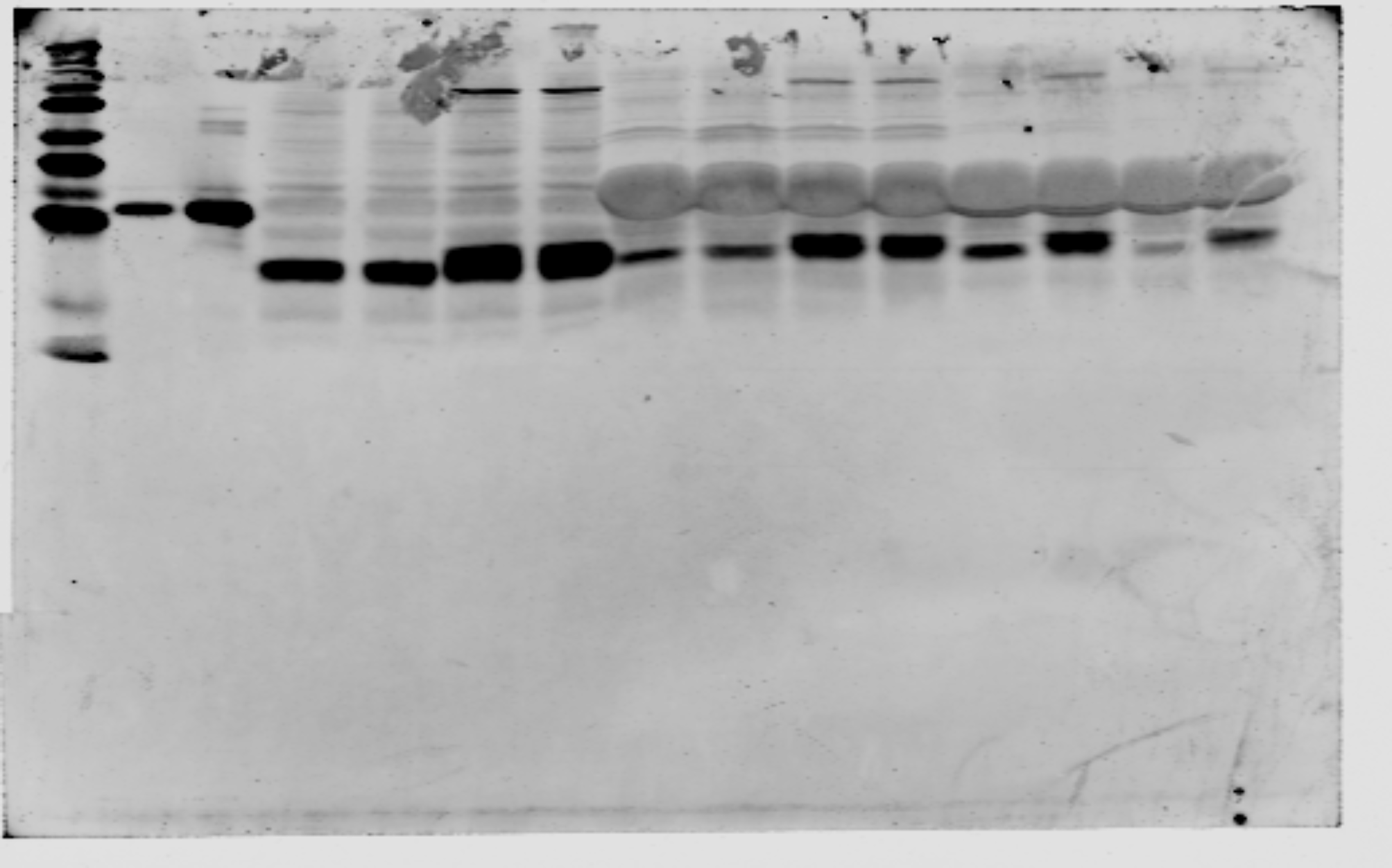

Supplement: Supplementary file 8 — Source Data Supplementary Figure 1c-part1 [file 41467_2019_10954_MOESM8_ESM.tif]

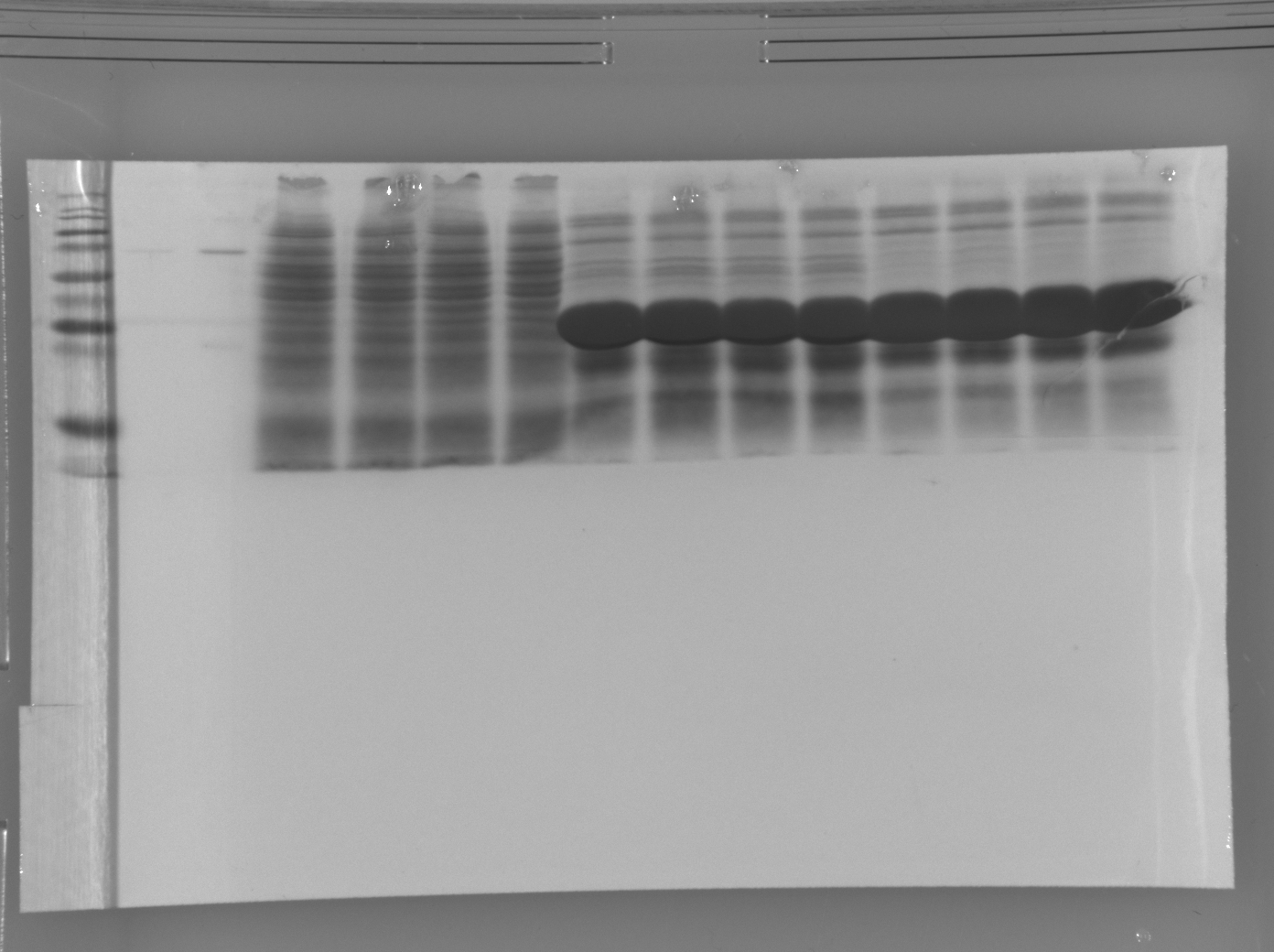

Supplement: Supplementary file 9 — Source Data Supplementary Figure 1c-part2 [file 41467_2019_10954_MOESM9_ESM.tif]
